# Physiological and pathological functions of LRRK2: implications from substrate proteins

**DOI:** 10.1042/NS20180005

**Published:** 2018-10-10

**Authors:** Miho Araki, Genta Ito, Taisuke Tomita

**Affiliations:** 1 Laboratory of Neuropathology and Neuroscience, Graduate School of Pharmaceutical Sciences, The University of Tokyo, 7-3-1 Hongo, Bunkyo-ku, Tokyo 113-0033, Japan; 2 Laboratory of Brain and Neurological Disorders, Graduate School of Pharmaceutical Sciences, The University of Tokyo, 7-3-1 Hongo, Bunkyo-ku, Tokyo 113-0033, Japan

**Keywords:** leucine rich repeat kinase, neurodegeneration, Parkinsons disease, rab

## Abstract

Leucine-rich repeat kinase 2 (LRRK2) encodes a 2527-amino acid (aa) protein composed of multiple functional domains, including a Ras of complex proteins (ROC)-type GTP-binding domain, a carboxyl terminal of ROC (COR) domain, a serine/threonine protein kinase domain, and several repeat domains. LRRK2 is genetically involved in the pathogenesis of both sporadic and familial Parkinson’s disease (FPD). Parkinson’s disease (PD) is the second most common neurodegenerative disorder, manifesting progressive motor dysfunction. PD is pathologically characterized by the loss of dopaminergic neurons in the substantia nigra pars compacta, and the presence of intracellular inclusion bodies called Lewy bodies (LB) in the remaining neurons. As the most frequent PD-causing mutation in LRRK2, G2019S, increases the kinase activity of LRRK2, an abnormal increase in LRRK2 kinase activity is believed to contribute to PD pathology; however, the precise biological functions of LRRK2 involved in PD pathogenesis remain unknown. Although biochemical studies have discovered several substrate proteins of LRRK2 including Rab GTPases and tau, little is known about whether excess phosphorylation of these substrates is the cause of the neurodegeneration in PD. In this review, we summarize latest findings regarding the physiological and pathological functions of LRRK2, and discuss the possible molecular mechanisms of neurodegeneration caused by LRRK2 and its substrates.

## Introduction

**Figure 1:**
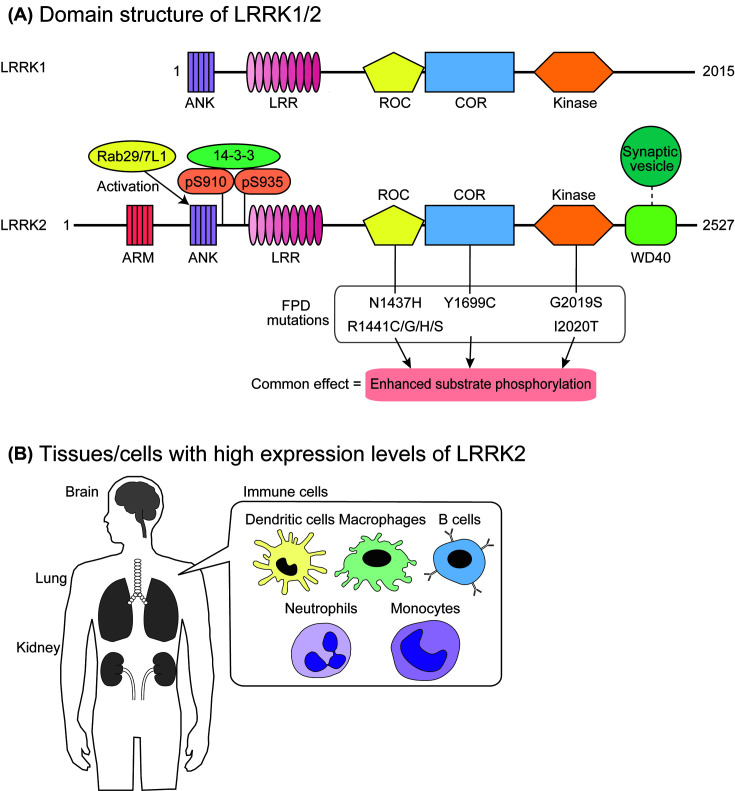
Structure and expression of LRRK2 (**A**) The domain structure of LRRK1/2. Both LRRK1 and LRRK2 are composed of an ROC-type GTP-binding domain, a COR domain, a serine/threonine protein kinase domain, and several repeat domains. Eight pathogenic mutations found in *LRRK2* are shown below the domain structure of LRRK2, which increase the levels of substrate phosphorylation *in vivo*. (**B**) LRRK2 is highly expressed in the brain, lungs, kidneys, and immune cells, including dendritic cells, macrophages, B cells, neutrophils, and monocytes.

Leucine-rich repeat kinase 2 (LRRK2) is a large protein consisting of 2527 amino acids (aa), harboring multiple functional domains, including a Ras of complex proteins (ROC)-type GTP-binding domain, a carboxyl terminal of ROC (COR) domain, and a serine/threonine protein kinase domain (Figure 1A). Proteins harboring both ROC and COR domains are categorized into the ROCO protein family, and LRRK2 was first described in the literature as Roco2 [1]. Most ROCO proteins have a kinase domain and some repeat domains besides the ROC and COR tandem domains [1]. There is a paralog of *LRRK2* in the mammalian genome, *LRRK1*, which produces a protein product with a similar domain structure to that of LRRK2 (Figure 1A).

LRRK2 has been implicated in the pathogenesis of Parkinson’s disease (PD). PD is one of the most common neurodegenerative diseases, and selectively affects dopaminergic neurons in the midbrain [2]. A pathological hallmark of PD is inclusion bodies called Lewy bodies (LBs), in which the major component is aggregated α-synuclein [3–5]. Although in most PD patients the disease is sporadic, there are several families in which PD is inherited, and this type of PD is called familial PD (FPD). The genetic linkage of the *PARK8* locus on chromosome 12q12 with one of the autosomal dominant forms of FPD was first identified in 2002 in a large Japanese family with multiple affected generations [6] (OMIM entry 607060), and several missense mutations in the *LRRK2* gene were subsequently identified from *PARK8*-linked FPD in 2004 [7,8]. To date, eight missense mutations (i.e. N1437H, R1441C/G/H/S, Y1699C, G2019S, and I2020T) in LRRK2 have been confirmed to be pathogenic [9] (Figure 1A). Moreover, several genome-wide association studies (GWAS) have demonstrated that single nucleotide polymorphisms located near the *LRRK2* locus are associated with the risk of sporadic PD [10–13]. Thus, mounting evidence suggests that LRRK2 plays an important role in the pathogenesis of PD.

In addition to dopaminergic neuronal loss, deposition of aggregated α-synuclein is one of the pathological hallmarks of PD brain. There have been a number of papers describing the histopathology of autopsied PD patients carrying LRRK2 mutations [14–20]. However, the α-synuclein pathology was surprisingly variable amongst mutation carriers, ranging from pure nigral degeneration without α-synuclein deposition, to massive deposition of α-synuclein in the cerebral cortex in addition to the midbrain. Thus, it remains unclear whether LRRK2 directly plays a role in the process of α-synuclein aggregation and deposition. Of note, LRRK2 has also been genetically implicated in several immunological disorders, namely inflammatory bowel diseases (IBDs), including Crohn’s disease and ulcerative colitis [21–23], Hansen’s disease (leprosy) [24,25], and systemic lupus erythematosus [26]. Collectively, given the unique domain structure of LRRK2 and its potential importance in the pathogenesis of several incurable diseases, it has received a large amount of attention from researchers. In this review, we systematically summarize our current understanding of the physiological roles of LRRK2 in the brain as well as in peripheral tissues, and discuss the possible mechanisms of how abnormalities in LRRK2 functions lead to the neurodegeneration observed in PD.

## Physiological roles of LRRK2

### Structure and function of domains in LRRK2

LRRK2 consists of 13 armadillo repeats (ARM; aa 49–657), 7 ankyrin repeats (ANK; aa 673–862), 14 leucine-rich repeats (LRR; aa 983-1319), an ROC domain (aa 1335–1515), a COR domain (aa 1515–1845), a protein kinase domain (aa 1859–2138), and 7 WD40 repeats (aa 2142–2498) [27–29] (Figure 1A). Amongst the FPD mutations, five mutations (i.e. N1437H, R1441C/G/H/S) are in the ROC domain, whereas Y1699C is in the COR domain, and G2019S and I2020T are in the kinase domain. In this section, we summarize the structure and function of these accessory domains apart from the kinase domain which will be discussed in a subsequent section in more detail.

The precise function of the ARM domains in LRRK2 is unclear. Based on a structural analysis of LRRK2 using cryo-electron microscopy (cryo-EM) and single particle analysis, LRRK2 forms a homodimer [30]. However, the ARM domain of LRRK2 extended out of the dimer core. Because ARM domains of other proteins (e.g. β-catenin) are involved in the protein–protein interaction [31], it might function in the association with other LRRK2-interacting proteins.

Amino acid substitutions of conserved leucines in the ANK domain of LRRK2 diminished the Rab29/7L1-mediated activation of LRRK2 [32]. Considering that the ANK domain in VPS9-domain ankyrin repeat protein (VARP) directly interacts with Rab32 [33], which is one of the closest homologs of Rab29/7L1, the ANK domain of LRRK2 might also directly interact with Rab29/7L1 (Figure 1A). It was also suggested that the ANK domain of LRRK2 intramolecularly interacts with the kinase domain of LRRK2 [34]. Guaitoli and colleagues [34] found that the model structure of the ANK and kinase domains of LRRK2 were similar to that of the kinase domain of cyclin-dependent kinase 6 (CDK6) and the inhibitory ankyrin domain of inhibitor of CDK4 (INK4) [35], suggesting that the ankyrin domain of LRRK2 is involved in the inhibition of its kinase activity.

The function of the LRR domain of LRRK2 is also not well understood. In a structure model of LRRK2, the LRR domain was on the surface of the LRRK2 dimer, suggesting the potential involvement of this domain in protein–protein interaction [34]. The amino-terminal region of the LRR domain in LRRK2 is known to be highly phosphorylated at Ser^860^, ^910^, ^935^, ^955^, and ^973^, amongst which the phosphorylations at Ser^910^ and Ser^935^ have been shown to be essential to the interaction with 14-3-3 proteins [36] (Figure 1A). Kinase(s) responsible for these phosphorylations remain elusive, whereas protein phosphatase 1 has been proposed as the responsible phosphatase for these sites [37]. However, it has been repeatedly shown that LRRK2 lacking the ARM, ANK, and LRR domains retains its intrinsic phosphotransferase activity in an *in vitro* system using peptide substrates [38,39]. Thus, it is clear that these domains are not involved in the intrinsic phosphotransferase activity of LRRK2.

In contrast with the other three repeat domains mentioned above, the WD40 domain seems to be essential to the intrinsic phosphotransferase activity of LRRK2 as LRRK2 lacking the WD40 domain did not phosphorylate the peptide substrate in an *in vitro* system [38]. An electron microscopic analysis has demonstrated that the WD40 domain of LRRK2 show doughnut-shaped particles, which is consistent with the typical structure of WD40 domains [40]. The authors also identified a large number of synaptic vesicle-associated proteins interacting with the WD40 domain of LRRK2 by MS, suggesting that the WD40 domain of LRRK2 plays an important role for controlling synaptic vesicle trafficking (Figure 1A).

The ROC domain of LRRK2 harbors motifs well-conserved amongst GTP-binding proteins. We and others have showed that GTP binding to the ROC domain of LRRK2 is essential to its intrinsic phosphotransferase activity [41,42]. It has also been shown that an LRRK2 mutant lacking the GTP-binding activity (i.e. T1348N) fails to phosphorylate Rab10, a physiological substrate of LRRK2, indicating that GTP binding to LRRK2 is required for eliciting its downstream signaling [32]. It is hypothesized that the ROC domain interacts with the kinase domain to activate it in a similar manner to the activation of Raf kinases by Ras [43]. In support of this notion, the FPD mutations in the ROC domain decrease its GTP-hydrolyzing activity and result in the increase in the GTP-bound form of LRRK2, thereby leading to the activation of the LRRK2 kinase activity [44,45]. In ROCO proteins, the ROC domain is always followed by a COR domain, forming a ROC-COR tandem domain [1], which suggests an important role of the COR domain related to the ROC domain. The existence of an FPD mutation (i.e. Y1699C) in the COR domain further underscores the importance of the COR domain in the LRRK2 function.

Of note, LRRK1, a paralog of LRRK2 with the similar ROC-COR-kinase domain structure, lacks the ARM and WD40 domains [27,46], which might result in differentiating the functions of two proteins from each other.

### Tissue distribution of LRRK2

LRRK2 is physiologically expressed in various tissues, with the highest expression in the brain, lung, and kidney [47] (Figure 1B). LRRK2 is ubiquitously expressed throughout the brain, but it has been suggested that its expression is relatively low in the substantia nigra and the ventral tegmental area of the midbrain where dopaminergic neurons are abundant [48–50]. In more recent studies using a defined set of specific antibodies against LRRK2, West and colleagues [52] found the highest expression of LRRK2 in the striatum, particularly in medium spiny neurons [51]. They also demonstrated the expression of LRRK2 in dopaminergic neurons in the substantia nigra. Although these studies using rodent brains were adequately performed using the brains of *Lrrk2* knockout (KO) mice as negative controls, the distribution of LRRK2 in the human brain remains largely unknown. *In situ* hybridization, quantitative real-time PCR, and immunohistochemical analysis have demonstrated that LRRK2 is expressed in regions affected in PD brains including the striatum and substantia nigra [53–55].

### Subcellular distribution of LRRK2

As LRRK2 does not contain obvious transmembrane domains, it is expected to be a cytosolic protein. Indeed, in cultured cells overexpressing LRRK2, LRRK2 is distributed throughout the cytoplasm but is not observed in the nucleus [56]. However, biochemical fractionation as well as careful immunocytochemical studies have suggested that LRRK2 also localizes to organelle membranes [56–59]. Immunoelectron microscopic analysis demonstrated that LRRK2 also localizes to various membranous structures [48]. Given the recent findings of the involvement of LRRK2 in membrane trafficking, it is possible that LRRK2 shuttles between the cytosol and membrane to regulate intracellular trafficking [60,61]. Intriguingly, Eguchi and colleagues [62] have recently discovered that LRRK2 is physiologically recruited on to the membranes of stressed lysosomes to maintain their homeostasis in co-operation with Rab29/7L1, Rab8, and Rab10. Moreover, LRRK2 often adopts a punctate localization at the perikarya, particularly when cells are treated with inhibitors for LRRK2 [63]. Kalogeropulou and colleagues [64] have demonstrated that this punctate structure is co-localized with p62/sequestosome-1, a ubiquitin-binding protein, although the nature of this punctate structure as well as whether this structure can be observed under physiological conditions remains unknown. The loss of phosphorylation at Ser^910^ and Ser^935^ of LRRK2, which are responsible for its interaction with 14-3-3 proteins, causes this translocation [63]. Although the biological significance of this phosphorylation-dependent translocation remains unknown, mouse embryonic fibroblasts (MEFs) endogenously expressing LRRK2 harboring the non-phosphorylated mutants S910A and S935A demonstrated reduced phosphorylation of Rab10 compared with wild-type MEFs [65]. Taken together, it would be reasonable to speculate that the phosphorylation at Ser^910^ and Ser^935^ promotes relocalization of LRRK2 to a subcellular compartment where LRRK2 efficiently phosphorylates Rab10. Identification of upstream kinase(s) responsible for the phosphorylation at Ser^910^ and Ser^935^ of LRRK2 will be important to elucidate the signaling pathway regulating the phosphorylation of Rab10.

### Physiological functions of LRRK2: implications from loss-of-function models

Several *Lrrk2* KO mouse/rat lines have been generated to date and have been extensively characterized to analyze the physiological roles of LRRK2 [66–73]. All these reports agree that the *Lrrk2* KO rodents are viable, fertile, and survive up to their usual lifespan with no obvious motor phenotypes, whereas they reproducibly found morphological abnormalities in various peripheral tissues, including the lungs and kidneys. Some reports described a significant increase in the number of hippocampal neuroblasts in *Lrrk2* KO animals, suggesting a role of LRRK2 in adult neurogenesis [72,74]. Collectively, these results indicate that LRRK2 does not play an essential role in animal survival, and that the loss-of-function of LRRK2 does not cause neurodegeneration, at least in rodents.

Morphological abnormalities have been reproducibly observed in the kidneys and lungs of some *Lrrk2* KO rodents [68,70,71,73,75]. The kidneys of *Lrrk2* KO animals show macroscopic enlargement and discoloration. Histological analyses demonstrated age-dependent accumulation of lipid-containing autofluorescent granules called lipofuscin and the autophagy substrate p62, in the renal tubules [68,70,73]. As lipofuscin granules are composed of peroxidized unsaturated fatty acids and are formed in lysosomes in aged animals, including humans [76], these results suggested that LRRK2 plays an important role in the autophagy-lysosomal pathway protecting renal tubules from age-dependent oxidative stress. The lungs of *Lrrk2* KO animals show specific enlargement of lamellar bodies in alveolar epithelial type II (ATII) cells [70,73,75]. ATII cells are specialized in the synthesis and secretion of pulmonary surfactant, which is released into the alveolar lumen upon the exocytosis of lamellar bodies [77]. Lamellar bodies display a unique concentric lamellar structure under the electron microscope and are thought to be a lysosome-related organelle, as they contain lysosomal markers [78]. Taken together, the peripheral phenotypes of *Lrrk2* KO animals suggest that LRRK2 deficiency compromises the homeostasis of lysosome-related organelles.

LRRK2 has a closely related ortholog known as LRRK1. To analyze whether LRRK1 can compensate for the loss of LRRK2 function, Giaime and colleagues [79] generated KO mice deficient in both *Lrrk1* and *Lrrk2* (*Lrrk1/2* double KO (DKO)) and discovered that the *Lrrk1/2* DKO mice show significant age-dependent neurodegeneration in areas relevant to PD. Immunohistochemical and biochemical analyses of *Lrrk1/2* DKO mice demonstrated an impairment of the autophagy-lysosomal pathway. These results imply that LRRK1 and LRRK2 have redundant functions in protecting neurons from degeneration. Toyofuku and colleagues [80] have generated *Lrrk1* KO mice, and they showed that *Lrrk1* KO mice exhibited neonatal lethality within 24 h after birth presumably due to lysosomal dysfunction. They further revealed that LRRK1 spatiotemporally regulates inactivation of Rab7A, thereby promoting the fusion of endocytic and autophagic components to lysosomes. LRRK1 has also been implicated in the endosomal trafficking of epidermal growth factor (EGF) receptors [81]. Collectively, these data suggest that LRRK1 and LRRK2 have redundant functions in the autophagy-lysosomal pathway. However, comparative proteomic analyses on the interactomes of LRRK1 and LRRK2 demonstrated little overlap between the two [82,83], which suggested that LRRK1 and LRRK2 might have distinct functions. Moreover, Taylor and colleagues [84] have shown that *LRRK1* variations are not a frequent cause of PD by sequencing the *LRRK1* gene in FPD patients. Nevertheless, further investigation would be required for clarifying the differences and similarities between the functions of LRRK1 and LRRK2.

LRRK2 might possess a scaffolding function through interaction with other proteins, as LRRK2 has multiple protein–protein interaction domains. Herzig and colleagues [70] generated knock-in mice harboring a kinase-dead (KD) mutation (D1994S) in LRRK2. These KD mice showed essentially the same phenotypes as the *Lrrk2* KO mice regarding kidney phenotypes, although they did not show any lung phenotypes. However, the expression levels of full-length LRRK2 were significantly decreased in the kidneys of KD mice. This observation again raised the possibility that reduction in the expression levels of LRRK2, but not the loss of its kinase activity, caused the kidney phenotype in D1994S knock-in mice. However, animals administered with LRRK2 inhibitors showed phenotypic changes at the kidneys and lungs in a similar manner to those observed in KO mice as discussed below. Thus, the scaffolding function of LRRK2, if any, does not seem to play an essential role in these phenotypes.

There have also been a number of reports describing the outcomes of LRRK2 inhibitor treatment in rodents [85,86]. Fell and colleagues utilized MitoPark [87] mice, which lack mitochondrial transcription factor A specifically in dopaminergic neurons, and showed the progressive degeneration of the nigrostriatal dopamine system. Although chronic oral administration of MLi-2, one of the most potent and selective inhibitors for LRRK2 [88], to MitoPark mice did not modify the progression of their behavioral and neurochemical phenotypes, MLi-2 administered mice showed similar morphological changes in the kidneys and lungs as have been observed in *Lrrk2* KO mice [85]. Andersen and colleagues chronically administered another potent LRRK2 inhibitor, PFE-360 [89], into rats and observed similar changes in the kidneys but not in the lungs [86]. These results suggested that the kinase activity of LRRK2 is responsible at least for the kidney phenotypes observed in *Lrrk2* KO mice. Fuji and colleagues [90] tested the peripheral effects of LRRK2 inhibitors in non-human primates. They administered GNE-7915, another potent LRRK2 inhibitor [91], to cynomolgus monkeys via oral gavage and found enlargement of lamellar bodies in lung ATII cells, similar to that observed in *Lrrk2* KO lungs. There were no morphological changes observed in the kidneys or in the brain in this monkey model.

The physiological consequences of the peripheral phenotypes observed in the loss-of-function models of LRRK2 have also been studied. *Lrrk2* KO mice were found to have proteinuria at the age of 18 months, which was not observed at the age of 2 months although they noticed the morphological changes including microvacuolization in *Lrrk2* KO kidneys even at the age of 6 weeks [70]. These mice also manifested breathing difficulties. In non-human primates administered with an LRRK2 inhibitor, a decrease in di-22:6-BMP, a biomarker of lysosomal dysregulation was observed [90]. Overall, the administration of LRRK2 inhibitors caused adverse effects due to peripheral inhibition, although a preliminary study suggested that the peripheral changes are reversible and do not cause pulmonary dysfunction [73]. However, there was a 16.5-month interval between the onset of morphological changes in the kidneys and the physiological dysfunction (i.e. proteinuria) in *Lrrk2* KO mice [70]. Thus, adverse effects on physiology caused by LRRK2 inhibitors might occur after several years of treatment also in humans. Future studies on the molecular mechanisms of these peripheral effects will help establish safer therapeutics based on LRRK2 inhibition.

### Substrate phosphorylation by LRRK2: downstream signaling

Since the discovery of the pathogenic mutations in *LRRK2*, a large amount of effort has been put into the identification of the physiological substrates of LRRK2. However, until recently, most substrates of LRRK2 reported are phosphorylated only in *in vitro* experimental settings [92]. For example, Jaleel and colleagues [38] identified the ERM family of proteins, including ezrin, moesin, and radixin, as *in vitro* substrates of LRRK2. Although the *in vitro* phosphorylation of ERM proteins by LRRK2 is reproducibly observed and the peptide sequence surrounding the phosphorylation site is now widely used as a peptide substrate for LRRK2 (i.e. LRRKtide; NH_2_–RLGRDKYK(T)LRQIRQ–COOH, where (T) stands for the threonine phosphorylated by LRRK2), the phosphorylation of ERM proteins by LRRK2 under physiological conditions has never been described. Nevertheless, LRRK2 phosphorylates serine or threonine, but not tyrosine, in substrate proteins as well as in substrate peptides [93], indicating that LRRK2 is a Ser/Thr kinase.

There is mounting evidence supporting the idea that LRRK2 phosphorylates tau. As GWAS of sporadic PD patients have repeatedly identified risk polymorphisms in the *MAPT* locus encoding tau proteins [11,12,94,95], and because PD patients with *LRRK2* mutations sometimes show tau accumulation [19,96,97], tau is thought to be one of the most pathologically relevant substrates. However, researchers have not yet reached a consensus regarding whether or not tau is physiologically phosphorylated by LRRK2. Screening for LRRK2 substrates have been carried out using fibroblast cells with low levels of tau expression [60]. Now that extensively validated research toolkits, such as knock-in mice harboring disease mutations, as well as highly specific brain-penetrable inhibitors for LRRK2 are available, it is important to address this question using mouse brains or primary neurons.

Apart from the physiological relevance, *in vitro* kinase assay experiments using artificial substrates of LRRK2 have provided important clues to elucidate the effects of the pathogenic mutations in LRRK2. Using the well-known non-specific substrate myelin-basic protein, West and colleagues [56] demonstrated that the most frequent pathogenic mutation, G2019S, increases the kinase activity of LRRK2 by two to three fold. However, the other mutations failed to reproducibly increase the *in vitro* kinase activity of LRRK2.

After several painstaking phosphoproteomic analyses, Steger and colleagues [60] finally identified physiologically relevant LRRK2 substrates, which were Rab proteins, including Rab8A/B and Rab10, and these were confirmed by a subsequent report [98]. To our surprise, whereas the G2019S mutation moderately increased the phosphorylation of Rab proteins *in vivo*, all other mutations, such as R1441G, also increased their phosphorylation by 10–20 fold [60,65]. The reason why the mutations other than G2019S increase LRRK2 kinase activity only *in vivo* remains unknown. This could be because the *in vitro* kinase assay lacks essential factor(s) required for the activation of LRRK2 by FPD mutations, or alternatively, FPD mutant forms of LRRK2 *in vivo* are more readily recruited to the place where LRRK2 becomes activated, resulting in higher levels of substrate phosphorylation (as discussed below).

Another intriguing LRRK2 substrate identified so far is p62/sequestosome-1 [64]. Kalogeropulou and colleagues [64] have shown that p62 binds to the ARM domain of LRRK2, and LRRK2 phosphorylates p62 at Thr^138^. p62 is a component of α-synuclein aggregates in PD brains [99]. It possesses a ubiquitin-binding domain as well as an LC3 interaction region (LIR), which sequestrates ubiquitinated proteins to autophagosomes for degradation. Park and colleagues [100] have also shown that p62 interacts with LRRK2 through the ARM/ANK domains of LRRK2. Given the involvement of LRRK2 in the autophagy-lysosomal pathway, it would be important to explore whether the p62 phosphorylation by LRRK2 plays a role in this pathway.

### Details of the phosphorylation of Rab proteins by LRRK2

In the initial report by Steger and colleagues [60], they proposed that the phosphorylation of Rab proteins by LRRK2 inhibits their interaction with GDP-dissociation inhibitors (GDIs) and guanine nucleotide exchange factors (GEFs), which has been reproduced in a subsequent study [101]. GDIs are essential for extracting GDP-bound forms of Rab proteins from their acceptor membranes, to recycle them back to donor membranes, and GEFs are required for the facilitation of GDP-GTP exchange (reviewed in [102]). Therefore, preventing Rab proteins from interacting with GEFs/GDIs should maintain them in their GDP-bound forms on the membrane.

**Figure 2:**
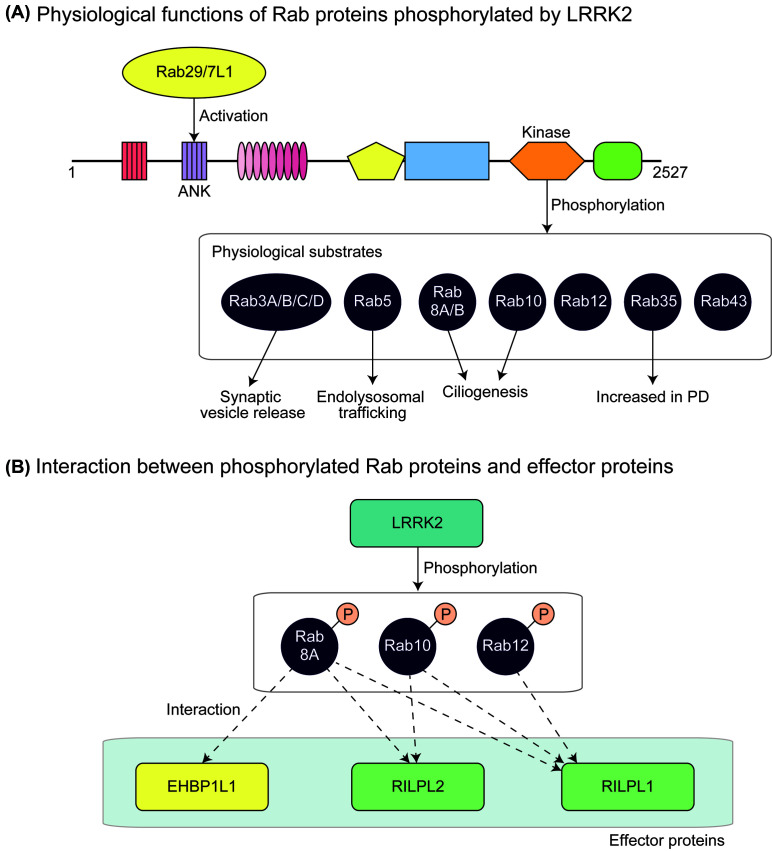
Physiological functions of Rab proteins phosphorylated by LRRK2 (**A**) Rab29/7L1 acts upstream of LRRK2, leading to its activation. Other Rab proteins are then endogenously phosphorylated by LRRK2. Rab3A/B/C/D are predominantly expressed in the brain and are involved in synaptic vesicle release. Rab8A/B and Rab10 are involved in endolysosomal trafficking. Rab8A/B and Rab10 have been shown to regulate primary ciliogenesis. Increased levels of Rab35 were detected in serum samples of PD patients compared with healthy controls, suggesting that Rab35 is involved in the pathogenesis of PD. (**B**) Phosphorylated Rab8A/B, Rab10, and Rab12 interact with RILPL1, whereas phosphorylated Rab8A and Rab10, but not Rab12, interact with RILPL2. It has also been shown that phosphorylated Rab8A binds to EHBP1L1.

Steger and colleagues [61] further elucidated that at least ten members of the Rab GTPase family, namely Rab3A/B/C/D, Rab8A/B, Rab10, Rab12, Rab35, and Rab43, are endogenously phosphorylated by LRRK2 under physiological conditions (Figure 2). Interestingly, these Rab proteins are closely associated with each other, as suggested by genomic analyses [103]. Importantly, the authors identified Rab-interacting lysosomal protein (RILP)-like 1 (RILPL1) and RILPL2 as specific interactors for p-Rab8A, Rab10, and Rab12. RILPL1 and RILPL2 belong to the RILP family sharing the amino-terminal RILP-homology 1 (RH1) and the carboxyl-terminal RILP-homology 2 (RH2) domains. As Rab8A/B, Rab10, and RILPL1/2 are reported to regulate primary ciliogenesis [104–106], they hypothesized that LRRK2 regulates primary ciliogenesis through the phosphorylation of these Rab proteins. Indeed, they showed that cells expressing FPD mutants of LRRK2 are deficient in primary ciliogenesis [61] (Figure 2). In a recent paper by Eguchi and colleagues [62], EH domain-binding protein 1 (EHBP1) and EHBP1-like protein 1 (EHBP1L1) were identified as effector proteins responsible for maintaining the homeostasis of stressed lysosomes. This is consistent with a previous finding showing that EHBP1L1 also binds to Rab8A in a phosphorylation-dependent manner [61]. It was also reported that, in hepatocytes, EHBP1 binds to both active Rab10 and EH domain-containing 2 (EHD2) to form Rab10–EHBP1–EHD2 complex under autophagy-stimulating conditions [107]. This complex is involved in engulfment of lipid droplets (LDs) by autophagic membrane. Therefore, it would be interesting to explore the possibility that LRRK2 regulates the engulfment of LDs via Rab10 phosphorylation. Collectively, it is tempting to speculate that LRRK2 recruited by Rab29/7L1 on to lysosomal membranes recruits and phosphorylates Rab8A/B and Rab10, thereby facilitating the interaction with their effector proteins, which might play a vital role on maintaining the homeostasis of lysosomes. In the future study, it is critical to pinpoint the responsible effector protein whose function is impaired in PD.

The phenotypes of KO mice of substrate Rab proteins are particularly important for discussing their physiological roles and the potential effects of their phosphorylation. Mice lacking *Rab10* were embryonically lethal at embryonic day 9.5 (E9.5). Embryos at E7.5 showed approximately 20% reduction in proliferating cells and large vacuoles in the cytoplasm, suggesting a marked inhibition of endosomal trafficking [108]. Mice lacking *Rab8a* showed microvillus atrophy, microvillus inclusions, and enlarged lysosomes in intestinal epithelial cells at 3 weeks of age, and died within 5 weeks after birth [109]. These phenotypes might be attributable to the mislocalization of apical proteins to lysosomes. DKO mice lacking both *Rab8a* and *Rab8b* showed essentially the same but exaggerated phenotypes compared with *Rab8a* single-KO mice, suggesting the redundant function of Rab8A and Rab8B [106]. Collectively, these results suggest that Rab8A/8B and Rab10 function in the endolysosomal pathway, which is consistent with the phenotypes observed in *Lrrk2* KO mice.

Both Rab8A/8B and Rab10 are reportedly involved in primary ciliogenesis [104], and *Rab8a/8b* DKO cells showed a significant deficiency in primary ciliogenesis only when *Rab10* was knocked down by RNAi [106]. Indeed, Steger and colleagues [61] reported that treatment of MEF with an LRRK2 inhibitor increased the number of ciliated cells. Given the proposed inhibitory function of Rab phosphorylation by LRRK2, these results are consistent with the view that LRRK2 plays an inhibitory role in ciliogenesis by phosphorylating the responsible Rab proteins. However, whether involvement of LRRK2 in ciliogenesis has any relevance to neurodegeneration in PD remains to be elucidated.

Rab3 has four isoforms, Rab3A, Rab3B, Rab3C, and Rab3D. Rab3A, Rab3B, and Rab3C are predominantly expressed in the brain and endocrine pituitary, whereas Rab3D is absent from the brain but is expressed in the endocrine pituitary, exocrine glands, and adipose tissues [110]. Schluter and colleagues [111] generated *Rab3* quadruple KO (QKO) mice lacking all four isoforms. *Rab3* QKO mice developed normally and were born alive but died shortly after birth because of respiratory failure. Cultured neurons from *Rab3* QKO mice showed a significant impairment in the release probability at synapses. To elucidate the effects of Rab3 phosphorylation by LRRK2 in neurons, it would be important to investigate whether cultured neurons from *Lrrk2* KO mice show a similar phenotype.

Although mice lacking *Rab12* have not been reported in the literature to our knowledge, *Rab12* KO mice have been generated by The International Mouse Phenotyping Consortium (IMPC) (https://www.mousephenotype.org/data/genes/MGI:894284). The IMPC website does not report an overt phenotype of *Rab12* KO mice in its phenotype database. In contrast, homozygous *Rab35* KO mice generated by the IMPC (https://www.mousephenotype.org/data/genes/MGI:1924657) are apparently embryonically lethal, while some eye phenotypes in heterozygous KO mice.

Whereas little is known about the causal association between Rab phosphorylation by LRRK2 and neurodegeneration in PD, Jeong and colleagues [112] found that the overexpression of Rab1A T75A mutant, Rab3C T94A mutant, as well as Rab35 T72A and T72D mutants caused TUNEL-positive apoptosis in primary neurons. The overexpression of Rab35 T72A and T72D mutants also caused neurodegeneration in mouse brains [112]. These results suggested that the phosphorylation of Rab35 plays an important role in the neurodegeneration observed in PD. Interestingly, in serum samples of PD patients, the levels of Rab35 were increased compared with healthy controls and patients with other neurodegenerative diseases (i.e. progressive supranuclear palsy (PSP) and multiple system atrophy) [113], further suggesting the possible involvement of Rab35 in the pathogenesis of PD.

### Regulation of LRRK2 by Rab proteins: upstream signaling events

Whereas Rab proteins are the physiological substrates of LRRK2, there has been accumulating evidence suggesting that LRRK2 is also regulated by Rab proteins. Amongst the genetic risk factors for idiopathic PD identified in GWAS was the genomic region including the *RAB29* (also known as *RAB7L1*) locus [10,11,13,95,114], and this region is assigned as *PARK16* (OMIM entry 613164). The association between *PARK16* and PD risk was subsequently validated in other ethnic populations [115], and in some reports, polymorphisms in the *RAB29* promoter region were found to be associated with PD risk [116], further underscoring the importance of *RAB29* in the pathogenesis of PD.

Meanwhile, MacLeod and colleagues [117] discovered a significant similarity between the transcriptome signatures of individuals harboring a PD risk allele either in *RAB29* or *LRRK2*, implicating a genetic interaction between these genes. Kuwahara and colleagues [118] further provided genetic evidence suggesting that in *Caenorhabditis elegans, RAB29* acts upstream of *LRRK2*. Importantly, they also showed that *Rab29* KO mice demonstrated abnormal lysosomal pathology in the kidneys, which is identical with the phenotype observed in *Lrrk2* KO mouse kidneys, supporting that the Rab29/7L1-LRRK2 axis also exists in mammals. Furthermore, there were reportedly no abnormalities in the brains of *Rab29* KO mice [118], suggesting that abnormal activation of the Rab29/7L1-LRRK2 axis is involved in the neurodegeneration occurring in PD.

More recently, three independent reports by Liu and colleagues [119], Purlyte and colleagues [32], and Fujimoto and colleagues [120] described that Rab29/7L1 activates LRRK2 in cells. They showed that Rab29/7L1 recruits LRRK2 to the trans-Golgi network (TGN), possibly via interaction with the ANK domain of LRRK2. They also showed that LRRK2 p-Rab29/7L1 on both Ser^71^ and Thr^72^, which apparently abolished the ability of Rab29/7L1 to activate LRRK2, suggesting negative feedback regulation of LRRK2 [32]. As the FPD mutant forms of LRRK2 were activated more robustly by Rab29/7L1 compared with wild-type LRRK2, the FPD mutant forms of LRRK2 have been hypothesized to show enhanced localization to the TGN, thereby becoming more readily activated by Rab29/7L1. Thus, genetic and biochemical evidence suggests that LRRK2 and Rab29/7L1 operate a common signaling pathway, and overactivation of this pathway is thought to be involved in the pathogenesis of PD.

### Physiological roles of LRRK2 in immune cells

In addition to the brain and peripheral tissues, LRRK2 is also highly expressed in immune cells, including B lymphocytes, monocytes, and neutrophils [121,122] (Figure 1B). In tissue culture models, LRRK2 protein expression was confirmed in murine bone-marrow derived macrophages, bone-marrow derived dendritic cells, the murine macrophage cell line RAW264.7, and the human monocytic cell line THP-1 [123–125]. It has also been reported that the expression of LRRK2 is induced in response to interferon-γ treatment in THP-1 cells as well as in human peripheral blood mononuclear cells, suggesting that LRRK2 plays a role in the innate immune system [123].

Notably, LRRK2 is genetically implicated in the pathogenesis of IBDs, including Crohn’s disease and ulcerative colitis [21–23]. In these reports, a genomic region containing the *LRRK2* and *MUC19* loci was identified as a susceptibility locus for IBD. Recently, Hui and colleagues [126] reported that variations in the *LRRK2* locus tend to have similar effects on the odds ratio of Crohn’s disease and PD. Thus, these results not only suggest the involvement of LRRK2 in the innate immune system but imply a common mechanism underlying IBD and PD [126].

Liu and colleagues [124] investigated the roles of LRRK2 in a mouse model of acute colitis induced by dextran sulphate sodium (DSS), which depends on the innate immune response. *Lrrk2* KO mice showed more severe response to DSS in their colons, including inflammatory infiltrates, thickened walls, and disruption of mucosal structures [124]. Another report from Zhang and colleagues [127] described the enhanced susceptibility of *Lrrk2* KO mice to intestinal infection, and suggested a possible role of LRRK2 in maintaining symbiosis with commensal bacteria to control intestinal infection. They found that in Paneth cells in the mouse intestine, LRRK2 localized to the membrane of dense core vesicles (DCVs), which store bactericidal substances, including lysozymes, and secrete them into the intestinal lumen upon infection. Importantly, lysozymes were missorted to lysosomes and degraded in Paneth cells from *Lrrk2* KO mice, leading to a deficiency of bactericidal activity. These results are in-line with the hypothesis that LRRK2 functions in regulating endolysosomal trafficking. Indeed, they found Rab10 as well as Rab2A localized to DCVs, although the involvement of phosphorylation-dependent regulation of Rab proteins is unclear at this stage. Although the molecular mechanisms are not well understood, these reports at least suggest that LRRK2 plays a role in the innate immune response.

Activation of microglia is a hallmark in the brain pathology of neurodegenerative diseases, including Alzheimer disease (AD) and PD (reviewed in [128]). Microglia are thought to be responsible for phagocytic activity in the brain (reviewed in [129]). LRRK2 has been shown to be expressed in activated microglia induced by an intracranial injection of lipopolysaccharide (LPS) to mouse brains [130]. LRRK2 is also expressed in primary cultured murine microglial cells [131]. *Lrrk2* KO primary microglia showed an attenuated inflammatory response to LPS treatment [132]. Daher and colleagues [133] showed that in rat brains, the administration of LPS as well as adeno-associated virus-mediated overexpression of human α-synuclein caused the selective degeneration of dopaminergic neurons in the substantia nigra, which was attenuated in *Lrrk2* KO rats. They also showed that, in the brains of *Lrrk2* KO rats, a smaller number of CD68-positive myeloid cells, including macrophages, monocytes, and microglia, were observed in the lesioned substantia nigra, suggesting an attenuated activation of the inflammatory response in *Lrrk2* KO rats. Consistent with this finding, primary microglia cultured from *Lrrk2* KO mice showed decreased interleukin 1β (IL-1β) production in response to LPS [132], which has also been observed in *Lrrk2* KO macrophages [134]. In this report by Liu and colleagues, they showed that the activation of inflammasomes containing NLR family CARD domain-containing protein 4 (NLRC4) in response to the infection of pathogens was insufficient in *Lrrk2* KO macrophages, leading to the reduced production of IL-1β [134]. Although the precise mechanisms of how LRRK2 regulates the activation of NLRC4-containing inflammasomes remain unclear, they suggested that the phosphorylation at Ser^533^ of NLRC4 by LRRK2 might be involved in this process.

Given the pathological evidence that inflammation plays an important role in the pathogenesis of PD (reviewed in [135]), it is possible to hypothesize that LRRK2 plays an important role in the innate immune response to pathogens under healthy conditions, but once overactivated, it has a deleterious effect on neurons through abnormal activation of the microglial inflammatory response.

## Pathological roles of LRRK2

### Histopathology of FPD patients harboring LRRK2 mutations

Neuropathological studies on PD patients harboring LRRK2 mutations have demonstrated surprisingly diverse pathology amongst patients, which has been summarized in a review by Schneider and Alcalay [136]. So far, many neuropathological studies have been conducted on autopsy samples from PD patients with *LRRK2* mutations, including N1437H [18], R1441C [8,14], R1441G [16,20], Y1699C [15], G2019S [97,137–142], and I2020T [17,19]. Although a loss of dopaminergic neurons in the substantia nigra was commonly observed amongst patients with *LRRK2* mutations, the accumulation of phosphorylated α-synuclein (e.g. LB and Lewy neurites), which is another hallmark pathology of PD, was not always observed [16,17]. Moreover, some patients showed an accumulation of phosphorylated tau as well as Transactive Response DNA binding protein 43 kDa (TDP-43). Although Kalia and colleagues [143] reported that LB pathology is more frequently associated with the G2019S mutation compared with the other mutations, Vilas and colleagues [97] reported two cases of G2019S carriers that did not show LB pathology and instead showed the accumulation of phosphorylated tau in astrocytes and neurons, which is a pathology consistent with PSP. Such diverse pathology was sometimes observed within the same family (e.g. a family with the R1441C mutation reported in [144], and a family with the I2020T mutation reported in [19]), suggesting that the pathological manifestations of PD patients harboring mutations in LRRK2 can be modulated by other genetic or environmental factors.

LRRK2 is genetically involved in the pathogenesis of both sporadic and FPD. Although FPD mutations in LRRK2 abnormally increase the phosphorylation of substrate Rab proteins, whether the phosphorylation of LRRK2 substrates is increased in sporadic PD has not been clarified. Maio and colleagues [145] recently reported that the LRRK2 kinase activity is increased in dopaminergic neurons in the brains of patients with idiopathic PD compared with healthy controls using a newly developed proximity ligation assay. Importantly, an immunohistochemical analysis demonstrated dramatically up-regulated Rab10 phosphorylation in PD brains, although the sample size was relatively small (eight controls compared with seven PDs). These results would encourage researchers to utilize the levels of Rab10 phosphorylation in peripheral samples for diagnosing as well as tracking PD. Recently, Fan and colleagues [122] reported the usefulness of peripheral blood neutrophils for quantitating the levels of Rab10 phosphorylation in human samples. This discovery opened up the possibility of investigating changes in Rab10 phosphorylation in PD patients, although a more high-throughput assay (e.g. ELISA) rather than immunoblotting would be required for undertaking a large-scale comparison between healthy elderly individuals and PD patients.

### Animals expressing FPD mutant forms of LRRK2

Since the discovery of pathogenic mutations in *LRRK2* that are linked with FPD, researchers have generated transgenic animals overexpressing LRRK2 in the brain or knock-in mice harboring disease-linked mutations in their endogenous *Lrrk2* gene, in the hope that such animals would model the disease and be useful for elucidating the molecular mechanisms of the disease (summarized in [146]). Transgenic rodents developed using bacterial artificial chromosomes (BACs) carrying wild-type or mutant forms of either the human or murine *LRRK2* gene showed a subtle but significant reduction in the level of striatal dopamine, although they failed to show nigral neurodegeneration or LB-like pathology [147–149]. Of note, these reports consistently described an increased staining of phosphorylated tau in the striatum.

Besides BAC-based transgenic animals, cDNA-based transgenic mice in which transgene expression is driven by an artificial promoter have been generated (summarized in [146]). Transgenic mice overexpressing G2019S LRRK2 under the cytomegalovirus enhancer and platelet-derived growth factor-β chain promoter showed significant loss of dopaminergic neurons at 12–16 months of age [150], which was not observed at younger ages [151]. On the other hand, another transgenic line overexpressing G2019S LRRK2 in dopaminergic neurons under the Pitx3 promoter in a tetracycline-dependent manner did not show nigral neurodegeneration, but again, they showed impaired release of dopamine in the striatum [152]. Considering the absence of neurodegeneration in BAC transgenic mouse lines, it has been controversial as to whether the phenotypes of cDNA-based transgenic mice have relevance to the pathophysiology of PD [146].

In this sense, knock-in mice harboring a mutation linked with FPD should be more relevant to physiological conditions in terms of the expression levels and the place where the transgenes are expressed. To date, none of the generated knock-in mice showed age-dependent neurodegeneration. However, again, Tong and colleagues [153] showed impaired dopaminergic transmission in R1441C knock-in mice and suggested impaired function of the dopamine D2 receptor. Furthermore, a more recent report by Yue and colleagues [154] described a significant reduction in the extracellular dopamine induced by amphetamine as well as an increase in the staining of phosphorylated tau in the striatum of G2019S knock-in mice. Overall, these results suggest that FPD mutant forms of LRRK2 impair dopaminergic transmission, although the underlying molecular mechanisms remain unknown.

In addition to the neuron-associated phenotypes, microglia-associated phenotypes have also been reported in LRRK2 transgenic rodents. BAC-based transgenic rats overexpressing human G2019S LRRK2 were more susceptible to LPS-induced nigral degeneration [155]. A larger number of CD68-positive myeloid cells were observed in the degenerated area in the brains of rats injected with LPS compared with non-transgenic rats. These results are consistent with the previous report from the same group showing opposite effects in *Lrrk2* KO mice [130]. However, such augmented responses to LPS were not observed in the recently reported BAC-based transgenic mice overexpressing human G2019S LRRK2 [156], and hence the effects of the overexpression of FPD mutant forms of LRRK2 on microglia remain controversial.

## Molecular mechanisms of neurodegeneration by LRRK2: hypotheses

**Figure 3:**
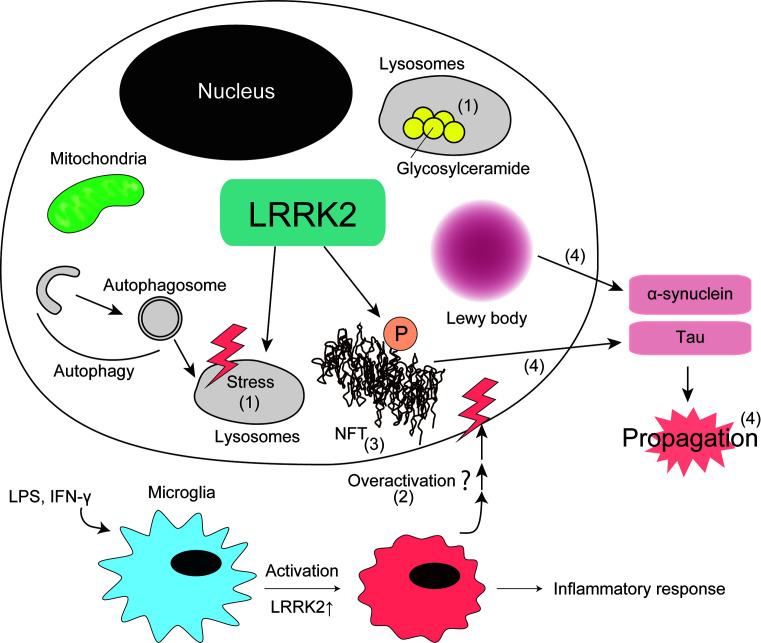
Pathological function of LRRK2 in PD Possible molecular mechanisms of PD pathology caused by LRRK2 based on the literature. (1) Lysosomal dysfunction, (2) microglia overactivation, (3) tau accumulation, and (4) prion-like propagation of aggregation-prone proteins.

In this section, we summarize hypotheses of how dysfunction of LRRK2 leads to neurodegeneration in PD based on the current understanding of physiological and pathological roles of LRRK2 (Figure 3).

### Lysosomal dysfunction

Considering that *Lrrk2* KO mice showed peripheral phenotypes associated with lysosomal dysfunction, it is reasonable to hypothesize that LRRK2 has some lysosome-associated functions also in neurons. Although neurons of *Lrrk2* KO mice appear to be normal, *Lrrk1/2* DKO mice showed age-dependent neurodegeneration and lysosomal dysfunction [79], suggesting that the lysosome-associated functions of LRRK2 is redundant, and can be compensated by LRRK1 in neurons. In this sense, it would be interesting to explore the possibility that there is a common lysosome-associated signaling pathway regulated by both LRRK1 and LRRK2 in neurons. In addition, considering the autosomal-dominant inheritance of *LRRK2* mutations, which suggests a gain-of-function mechanism, it would be important to investigate whether the overactivation of LRRK2 also causes the dysfunction of lysosomes.

The involvement of lysosomal dysfunction in the pathogenesis of PD is also supported by two genetic findings. First, loss-of-function mutations in the *ATP13A2* gene, which is also called *PARK9*, have been found in families with a rare hereditary form of juvenile-onset parkinsonism, called Kufor–Rakeb syndrome (reviewed in [157]). ATP13A2 is a lysosomal P-type ATPase [158], and a cell study has suggested that ATP13A2 is involved in the homeostasis of intracellular Zn^2+^ [159]. As compromising Zn^2+^ homeostasis leads to impaired lysosomal function, it has been strongly suggested that *PARK9*-type parkinsonism is caused by lysosomal dysfunction.

Another line of evidence of lysosomal involvement in the pathogenesis of sporadic PD is from etiological studies of Gaucher’s disease (GD), showing a high prevalence of PD in GD patients [160]. GD is an autosomal recessive lysosomal storage disorder caused by mutations in the *GBA* gene encoding glucocerebrosidase (GCase). GCase is a lysosomal enzyme responsible for the degradation of glucocerebroside to glucose and ceramide. *GBA* mutations decrease GCase activity, leading to the accumulation of glucocerebroside in lysosomes, which in turn compromises lysosomal activity (reviewed in [161]). More recently, Sidransky and colleagues [162] undertook a large-scale genetic study and reported a strong association between *GBA* mutations and sporadic PD.

Collectively, it is most likely that the overactivation of LRRK2 compromises lysosomal homeostasis, thereby leading to the neurodegeneration observed in PD.

### Overactivation of microglia

There have been a large number of reports describing an increase in blood cytokine levels in PD patients as well as in PD model animals (reviewed in [163]), suggesting that inflammation plays a role in the pathogenesis of PD. Given that the expression of LRRK2 has been shown to be up-regulated in response to LPS and inflammatory cytokines, it is likely that LRRK2 is involved in the innate immune response in the brain as well as peripheral tissues.

Given that *Lrrk2* KO mice show vulnerability to experimentally induced colitis as well as to intestinal infection, LRRK2 might play an important role in maintaining the innate immune system active in peripheral tissues. LRRK2 may also play a role in activating microglia in the brain, as suggested by rodent models injected with LPS to elicit neuroinflammation. Therefore, although systemic activation of LRRK2 might be beneficial outside the brain in terms of protection from bacterial infection, it also causes the overactivation of microglia in the brain, which can somehow cause deleterious effects on neurons. Indeed, it was reported very recently that chronic inflammation in the periphery can modulate neuropathology in the brain in a mouse model of AD [164]. Therefore, maintaining normal LRRK2 activity might be important for balancing the peripheral demand for innate immunity and the overactivation of microglia in the brain.

### Abnormal tau accumulation

Accumulation of abnormally phosphorylated tau, a microtubule-associated protein, is a hallmark pathology of several neurological disorders, including AD, frontotemporal dementia, Pick disease, and corticobasal degeneration (reviewed in [165]). Although GWAS indicate that tau is also involved in the pathogenesis of sporadic PD besides α-synuclein and LRRK2 [11,13], its precise role has not been well understood.

As mentioned above, a number of reports have described that LRRK2 promotes tau phosphorylation in the brain in BAC-based transgenic mice as well as in knock-in mice of FPD mutant forms of LRRK2. Whereas the direct phosphorylation of tau by recombinant LRRK2 in an *in vitro* assay has been observed [166], some reports suggested that LRRK2 phosphorylates tau in the presence of tubulin or microtubules [167,168]. Other reports proposed that LRRK2 directly interacts with GSK-3β via the LRRK2 kinase domain, and this interaction enhances the kinase activity of GSK-3β [169]. In this report, the phosphorylated GSK-3β phosphorylates tau at Ser^396^. tau phosphorylation at Ser^396^ was elevated in cells overexpressing LRRK2 G2019S compared with those overexpressing WT LRRK2. More recently, Ohta and colleagues [170] also showed that induced pluripotent stem (iPS) cells established from PD patients carrying I2020T mutation showed activation of GSK-3β and high levels of tau phosphorylation. Lin and colleagues [171] have reported that, in fruitfly neurons, overexpression of LRRK2 G2019S promoted the recruitment of activated Shaggy, a fly homolog of GSK-3β, to dendrites, leading to accumulation of hyperphosphorylated tau in dendrites and dendrite degeneration. Shanley and colleagues [172] have found that LRRK2 strongly binds to tau *in vitro* and that LRRK2 is co-immunoprecipitated with tau and Cdk5, the latter of which is one of the major protein kinases phosphorylating tau besides GSK-3β. Based on these observations, they suggested that LRRK2 might serve as a scaffold protein, facilitating the phosphorylation by Cdk5. It has also been shown that microtubule affinity-regulating kinase 1 (MARK1) also directly interacts with LRRK2 and phosphorylated by LRRK2 at Thr^215^ and Ser^219^ *in vitro* and in cells. MARK1 plays an important role in regulating microtubule stability through phosphorylation of microtubule-bound tau [173]. Collectively, it remains possible that LRRK2 recruits or activates tau kinases, such as GSK-3β, Cdk5, and MARK1, to indirectly promote tau phosphorylation. Therefore, it remains controversial as to whether LRRK2 directly or indirectly promotes tau phosphorylation.

Some, but not all PD patients harboring *LRRK2* mutations manifested tau accumulation in the brain [15,19]. However, tau pathology is also found in healthy elderly controls [174], which makes it difficult to conclude the causal association between the tau accumulation observed in PD patients harboring *LRRK2* mutations and neurodegeneration.

### Prion-like propagation of PD-associated proteins

It has been experimentally established that intracellularly aggregated proteins, including α-synuclein and tau, are transferred from cell to cell (reviewed in [175]). This transmission is generally called ‘prion-like propagation’, as the spreading of abnormally accumulated proteins in the brain resembles prion diseases (reviewed in [176]).

It has been pointed out that the histopathological manifestations in the brains of PD patients harboring FPD mutations in LRRK2 are pleiotropic, ranging from pure nigral degeneration without α-synuclein deposition [16,17,20], to dementia with LB (DLB)-like widespread α-synuclein pathology [8,15]. Although the mechanism underlying this prion-like propagation of α-synuclein is still controversial, it would be reasonable to conceive that the process involves intracellular trafficking for transporting aggregated proteins out of cells and for taking them up into cells. Therefore, it is possible that LRRK2 is involved in the propagation mechanisms, given its potential role in the regulation of Rab proteins. The first simple but fundamental experiment would be to investigate the propagation of α-synuclein and in *Lrrk2* KO mice, as well as in mice treated with LRRK2 inhibitors. If the loss-of-function of LRRK2 attenuates the propagation, then identification of responsible substrates of LRRK2 would be the next step. Considering the proposed functional redundancy between LRRK1 and LRRK2 in the brain, it would also be interesting to test these hypotheses in *Lrrk1/2* DKO mice.

## Conclusion

This review aimed at providing a systematic overview of the current understanding of the physiological functions of LRRK2, to discuss how LRRK2 causes PD. Although it has been 14 years since the discovery of the pathogenic mutations in *LRRK2*, research on the physiological functions of LRRK2 has only just begun, particularly in the context of its involvement in intracellular membrane trafficking. Further investigation into the details of the functions of LRRK2 should be conducted to establish mechanism-based therapies for PD, which currently remains incurable.

## Abbreviations

AD: Alzheimer disease
ATII: alveolar epithelial type II
BAC: bacterial artificial chromosome
COR: carboxyl terminal of ROC
DCV: dense core vesicle
DKO: double knockout
DSS: dextran sulphate sodium
EHBP1: EH domain-binding protein 1
EHBP1L1: EHBP1-like protein 1
EHD2: EH domain-containing 2
FPD: familial Parkinson’s disease
GCase: glucocerebrosidase
GD: Gaucher’s disease
GDI: GDP-dissociation inhibitor
GEF: guanine nucleotide exchange factor
GWAS: genome-wide association study
IBD: inflammatory bowel disease
IL-1β: interleukin 1β
IMPC: International Mouse Phenotyping Consortium
KD: kinase-dead
KO: knockout
LB: Lewy body
LPS: lipopolysaccharide
LRRK1/2: leucine-rich repeat kinase 1/2
MEF: mouse embryonic fibroblast
NLRC4: NLR family CARD domain-containing protein 4
PD: Parkinson’s disease
PSP: progressive supranuclear palsy
QKO: quadruple KO
RILP: Rab-interacting lysosomal protein
RILPL1/2: RILP-like protein 1/2
ROC: Ras of complex protein
